# Increased YKL-40 but Not C-Reactive Protein Levels in Patients with Alzheimer’s Disease

**DOI:** 10.3390/biomedicines9091094

**Published:** 2021-08-27

**Authors:** Víctor Antonio Blanco-Palmero, Marcos Rubio-Fernández, Desireé Antequera, Alberto Villarejo-Galende, José Antonio Molina, Isidro Ferrer, Fernando Bartolome, Eva Carro

**Affiliations:** 1 Network Center for Biomedical Research in Neurodegenerative Diseases (CIBERNED), 28031 Madrid, Spain; victorb1989@gmail.com (V.A.B.-P.); marcosrubio.imas12@h12o.es (M.R.-F.); eeara@yahoo.es (D.A.); avgalende@yahoo.es (A.V.-G.); cvillaiza@telefonica.net (J.A.M.); 8082ifa@gmail.com (I.F.); 2 Group of Neurodegenerative Diseases, Hospital 12 de Octubre Research Institute (imas12), 28041 Madrid, Spain; 3 Neurology Service Hospital Universitario 12 de Octubre, 28041 Madrid, Spain; 4 Bellvitge Biomedical Research Institute (IDIBELL), Hospitalet de Llobregat, E08907 Barcelona, Spain; 5 Department of Pathology and Experimental Therapeutics, University of Barcelona, E08900 Barcelona, Spain; 6 Institute of Neurosciences, University of Barcelona, E08000 Barcelona, Spain

**Keywords:** Alzheimer’s disease, Parkinson’s disease, YKL-40, C-reactive protein, CSF and plasma biomarkers, inflammation, astrogliosis

## Abstract

Neuroinflammation is a common feature in Alzheimer’s (AD) and Parkinson’s (PD) disease. In the last few decades, a testable hypothesis was proposed that protein-unfolding events might occur due to neuroinflammatory cascades involving alterations in the crosstalk between glial cells and neurons. Here, we tried to clarify the pattern of two of the most promising biomarkers of neuroinflammation in cerebrospinal fluid (CSF) in AD and PD. This study included cognitively unimpaired elderly patients, patients with mild cognitive impairment, patients with AD dementia, and patients with PD. CSF samples were analyzed for YKL-40 and C-reactive protein (CRP). We found that CSF YKL-40 levels were significantly increased only in dementia stages of AD. Additionally, increased YKL-40 levels were found in the cerebral orbitofrontal cortex from AD patients in agreement with augmented astrogliosis. Our study confirms that these biomarkers of neuroinflammation are differently detected in CSF from AD and PD patients.

## 1. Introduction

Neuroinflammation is now widely accepted as a pathological hallmark of Alzheimer’s (AD) [1,2] and Parkinson’s (PD) [3,4,5] disease. Several damage signals appear to induce neuroinflammation, including β-amyloid (Aβ) oligomers, tau, and α-synuclein (α-syn), mediated by the progressive astrocyte and microglial cell activation with the consequent overproduction of proinflammatory agents that may leak toward cerebrospinal fluid (CSF) [6]. Despite the analysis of these agents in CSF being a tempting topic to study, levels of inflammatory markers in CSF from AD and PD patients have not been sufficiently investigated. A standard clinical application of inflammatory markers in the clinical diagnosis of these neurodegenerative disorders is lacking, likely owing to contradictory and heterogeneous findings of numerous studies [7,8].

Among these neuroinflammatory markers found in biological samples is YKL-40 (also named Chitinase 3-like I). This marker has been largely associated with the pathogenesis of a variety of human diseases, many of them sharing chronic inflammatory features and high cellular activity, including rheumatoid arthritis, hepatic fibrosis, and asthma, where YKL-40 levels were found elevated in patient peripheral blood [9,10,11]. YKL-40 is a secreted glycoprotein with functions including tissue remodeling during inflammation and angiogenic processes, which make YKL-40 a good marker of inflammation and endothelial dysfunction [12,13,14]. YKL-40 was found elevated in CSF from several acute and chronic neuroinflammatory conditions [15], as well as in preclinical and prodromal AD/mild cognitive impairment (MCI) [16,17,18]. This is consistent with the potential role of astrocytosis in early AD pathogenesis [19] and with the fact that *YKL-40* expression and YKL-40 protein levels are abundant in reactive astrocytes and residual in microglial cells [15,20,21]. Additionally, YKL-40 was found close to amyloid plaques and neurofibrillary tangles in AD [16]. Contrarily, other works reported different results showing no significant differences in YKL-40 levels in CSF from MCI and AD patients compared with cognitively normal subjects [22]. Other works indicated increased CSF YKL-40 levels only in AD but not in MCI subjects compared with healthy controls [23,24]. Regarding PD, YKL-40 concentrations in CSF were found either decreased or unchanged [25,26].

Although YKL-40 can be considered one of the most promising neuroinflammatory biomarkers in AD, the abovementioned works indicate that brain YKL-40 levels patterns in different neurodegenerative diseases and the potential correlation between brain and CSF levels is largely unknown, indicating that more research regarding YKL-40 expression pattern is required.

On the other hand, C-reactive protein (CRP), a kind of acute-phase protein regulated by proinflammatory cytokines, is the most studied biomarker of systemic inflammation [27]. CRP was linked to chronic inflammatory and neurodegenerative diseases, such as AD and PD [28]. Elevated CRP peripheral blood levels have been frequently associated with increased risk of dementia and cognitive decline. Studies carried out investigating the association between markers of inflammation and risk of dementia showed conflicting results. A systematic review and meta-analysis found that elevation of peripheral CRP levels was associated with increased risk of developing dementia [29]. Nevertheless, another meta-analysis found no significant differences in serum CRP levels between patients with AD and healthy subjects [30]. Epidemiological studies have also explored the relationship between CRP levels and AD risk, describing lower CRP levels in CSF from AD patients [31,32]. Regarding PD and CRP levels, results in the literature are still contradictory. A significant increase in blood CRP levels was reported in subjects suffering from PD compared with healthy controls [33,34], while other works did not identify such a tendency, instead reporting no differences [35]. Furthermore, the CRP levels in CSF remained unchanged in PD patients when compared with healthy subjects [26,32]. Despite these differences, CRP is considered a prominent “risk factor” for PD [36].

Growing evidence indicates that blood-borne CRP can cross the blood–brain and blood–spinal cord barriers; thus, CRP can be found in the CSF and deposited in the diseased central nervous system (CNS). The source of CRP might also be local. However, CRP production may occur in multiple CNS-resident cells including neurons, microglia, and astrocytes [37,38,39]. Regardless of its origin (hepatic versus local), the presence of CRP in the CNS is associated with numerous diseases including AD [40]. CRP levels were also found increased in brain parenchyma tissue after intracerebral hemorrhage [41]. Additionally, large amounts of the protein were present in perihematomal regions and within neurons and glia of patients who died within 12 h of spontaneous intracerebral hemorrhage [41,42].

Despite these accumulative data supporting a role of neuroinflammation, particularly YKL-40 and CRP in AD and PD, there is no definitive evidence reflecting the peripheral (blood) and central (CSF) concentration changes of YKL-40 and CRP in AD and/or PD patients. We think that further research is needed to elucidate the variable pattern of these inflammatory biomarkers in the CSF and blood from AD and PD patients. In this work, we aimed at clarify YKL-40 and CRP concentrations measured in CSF and plasma and to determine their specificity in AD and PD. To address this issue, we analyzed YKL-40 and CRP levels in CSF and plasma from a well-characterized cohort of patients with MCI, AD, and PD, using sensitive enzyme-linked immunosorbent assays (ELISAs).

## 2. Material and Methods

### 2.1. Human Donors

A total of 123 subjects were included in this study: (1) elderly nondemented subjects without any evidence of any neurodegenerative disease (healthy controls) classified as controls (*n* = 37); (2) MCI due to AD (MCI) patients (*n* = 22); (3) probable mild/moderate–severe sporadic AD patients (*n* = 34); (4) PD patients (*n* = 30). Study participants were enrolled from the Memory Clinic (controls, MCI and AD subjects) and Movement Disorders Unit (PD participants) of Hospital Universitario 12 de Octubre (Madrid, Spain). Subject demographic and clinical characteristics are listed in Table 1.

All participants were classified using established diagnostic criteria into those with MCI or probable AD dementia [43,44,45]. Diagnosis was based on detailed clinical assessment, neuropsychological evaluation, and neuroimaging (MRI). Functional impairment was measured via the Clinical Dementia Rating (CDR) score [46]. PD patients were diagnosed following the Movement Disorder Society (MDS) clinical diagnostic criteria [47], and all fulfilled criteria for clinically established PD. PD patients did not refer cognitive complaints and did not exhibit symptoms of dementia. The control group was constituted by cognitively normal individuals aged 50 years or older, without clinical signs of cognitive impairment and without neurological or psychiatric disease history. Exclusion criteria for every participant were concomitant significant cerebrovascular disease and evidence of any neurological, psychiatric, medication, or non-neurological medical comorbidity that could affect cognition or motor function.

Approval of the study was obtained from the Research Ethics Committee of Hospital Universitario 12 de Octubre, and all participants provided written informed consent.

### 2.2. Fluid Sample Collection

CSF samples were collected from all subjects (including healthy patients and MCI, AD, and PD subjects) and processed according to standardized procedures by lumbar puncture in 15 mL sterile polypropylene tubes. Samples were then centrifuged at 3000 rpm at 4 °C for 10 min. Supernatant aliquots were stored at −80 °C into 0.5 mL polypropylene cryogenic tubes with Protease Inhibitor Cocktail (Roche, Basel, Switzerland). 

Blood samples were obtained through antecubital vein puncture from patients and healthy subjects. Plasma was isolated from whole blood collected in 7 mL EDTA-2Na tubes. Whole blood was centrifuged at 2000 rpm for 10 min at room temperature. Supernatants were then collected and aliquoted in polypropylene cryogenic tubes with Protease Inhibitor Cocktail (Roche, Basel, Switzerland) and stored at −80 °C.

### 2.3. Tissue Samples

Postmortem cerebral orbitofrontal cortex tissue was obtained from brain donors diagnosed with AD and control individuals. Frozen samples were supplied by the Institute of Neuropathology Brain Bank IDIBELL-Hospital Universitari de Bellvitge (Hospitalet de Llobregat, Spain). Subject consent was obtained according to the Declaration of Helsinki, and approval came from the Research Ethics Committee of the responsible institution. For all cases, written informed consent was available. Subjects were selected on the basis of postmortem diagnosis of AD according to neurofibrillary tangle pathology and Aβ plaques [48]. AD cases showed high AD neuropathologic change (Braak stage V/VI and moderate to frequent neuritic plaque score). Control participants were considered those with/without neurological symptoms or a low grade of AD neuropathologic change. A total of 24 samples were categorized into AD and controls, as presented in Table 2.

### 2.4. DNA Purification and Apolipoprotein E (APOE) Genotyping

Genomic DNA was extracted from peripheral blood using QIAmp DNA Blood Mini Kit (Qiagen, Hilden, Germany), according to the manufacturer’s instructions. Human APOE C112R and R158C polymorphisms were detected to identify the APOE ε2, ε3, and ε4 alleles, using the LightCycler 480 II Instruments Kit (Roche Diagnostics, Basel, Switzerland) following manufacturer instructions.

### 2.5. Protein Analysis

CSF and plasma concentrations of the neuroinflammatory biomarkers (YKL-40 and CRP) were analyzed using ELISA kits (Human Chitinase 3-like 1 Quantikine ELISA kit (DC3L10), R&D; Human CRP Quantikine ELISA kit (DCRP00), R&D) according to the manufacturer’s instructions.

Brain YKL-40 and GFAP protein levels were also examined by Western blotting. Postmortem cerebral orbitofrontal cortex tissue was obtained from brain donors diagnosed with AD and control individuals. Briefly, human cerebral orbitofrontal cortex samples were incubated and homogenized in lysis buffer (50 mM Tris/HCl buffer, pH 7.4 containing 2 mM EDTA, 0.2% Nonidet P-40, 1 mM PMSF, Protease and Phosphatase Inhibitor Cocktails; Roche, Basel, Switzerland) and centrifuged for 10 min at 14,000 rpm at 4 °C. Supernatants were recovered and stored at −80 °C. Protein content was determined using the BCA method (Thermo Fisher Scientific, MA, USA). Equal amounts of protein (20 µg for YKL-40 and 5 µg for GFAP) were mixed with Laemmli sample buffer supplemented with β-mercaptoethanol, heated to 95 °C for 5 min, resolved by 10% NuPAGE Bis-Tris Gels (Thermo Fisher Scientific, MA, USA), and transferred onto polyvinylidene difluoride (PVDF) membranes (Millipore, MA, USA). Afterward, membranes were blocked and incubated overnight at 4 °C with primary antibodies: a recombinant rabbit monoclonal anti-YKL-40 antibody (ab255297, 1:500, Abcam) and a mouse monoclonal anti-GFAP antibody (G3893, 1:0000, Sigma Aldrich). Membranes were then incubated for 1 h with the appropriate horseradish peroxidase (HRP)-conjugated secondary antibodies (G-21234, 1:5000, Thermo Fisher Scientific, MA, USA; ab97023, 1:40000, Abcam). Protein loading was monitored using mouse monoclonal HRP-conjugated antibodies against α-tubulin (ab40742, 1:5000, Abcam) for YKL-40 or against β-actin (A1978, Sigma Aldrich) for GFAP detection. Immunocomplexes were revealed by an enhanced chemiluminescence reagent (ECL Clarity; Bio Rad, CA, USA). Densitometric quantification was carried out with Image Studio Lite 5.0 software (Li-COR Biosciences, NE, USA). Protein bands were normalized to loading controls and expressed as a percentage of the control group. 

### 2.6. Statistical Analysis

Statistical analysis and graphs were performed using Stata/IC software (Stata 16.1, StataCorp LLC, College Station, TX, USA) and Prism (GraphPad Software version 8.00, La Jolla, CA, USA). After assessing the normality of the distribution, differences in CSF and plasma YKL-40 and CRP levels between groups were analyzed using the nonparametric Kruskal–Wallis rank test. The *p*-value for pairwise comparisons is displayed with Bonferroni correction. A descriptive multiple linear regression model was performed to account for confounding variables (age, sex, and APOE ε4) in CSF YKL-40 association analysis. Interactions of confounding variables with the clinical diagnosis were excluded from the model (significance for the whole set of interactions: *p* > 0.10). The regression coefficient is displayed as “b”. Differences in sex distribution, age of participants, age at onset, and years since the onset of the disease between groups were evaluated with Pearson chi-squared and ANOVA tests, where appropriate. Associations between biomarkers and demographic characteristics were examined with Pearson correlation tests, Student *t*-tests and Mann–Whitney U tests, where appropriate. A nonparametric trend test (Jonckheere trend test) was performed to evaluate the existence of a trend when the exposition showed ordinal categories. ROC curves were constructed after modeling the presence or absence of a given clinical diagnosis with a regression logistic analysis. YKL-40 and GFAP Western blot expression levels were normalized to their respective loading controls (α-tubulin and β-actin) and compared with the mean of the control ratio with the nonparametric Mann–Whitney U test. In graphs, CSF and plasma YKL-40 and CRP levels are shown as median and interquartile range. The brain expression of YKL-40 is shown as the mean ± standard error of the mean (SEM). In all cases, statistical significance was set at *p* < 0.05.

## 3. Results

### 3.1. Associations with Demographic Data

Demographic and clinical data are shown in Table 1 for further characterization of the study cohort. A total of 34 subjects were clinically diagnosed with AD, 22 subjects were grouped as MCI, and 30 subjects were diagnosed with PD. Individuals diagnosed with AD were slightly older than the rest of the cohort, including PD, MCI, and healthy subjects. Female sex was overrepresented in the AD and MCI groups, while males represented around 60% of controls and PD subjects. APOE ε4 carriers were more prevalent in the MCI/AD group than in controls, according to previous publications [49]. Most AD patients had clinically mild dementia (74% scored 1 in CDR scale), and none of the PD patients reached the dementia stage. Furthermore, the majority of individuals diagnosed with PD exhibited mild motor impairment (73% of them were in Hoehn & Yahr stage 1 or 2).

### 3.2. YKL-40 and CRP Levels in Different Diagnostic Groups

**Figure 1:**
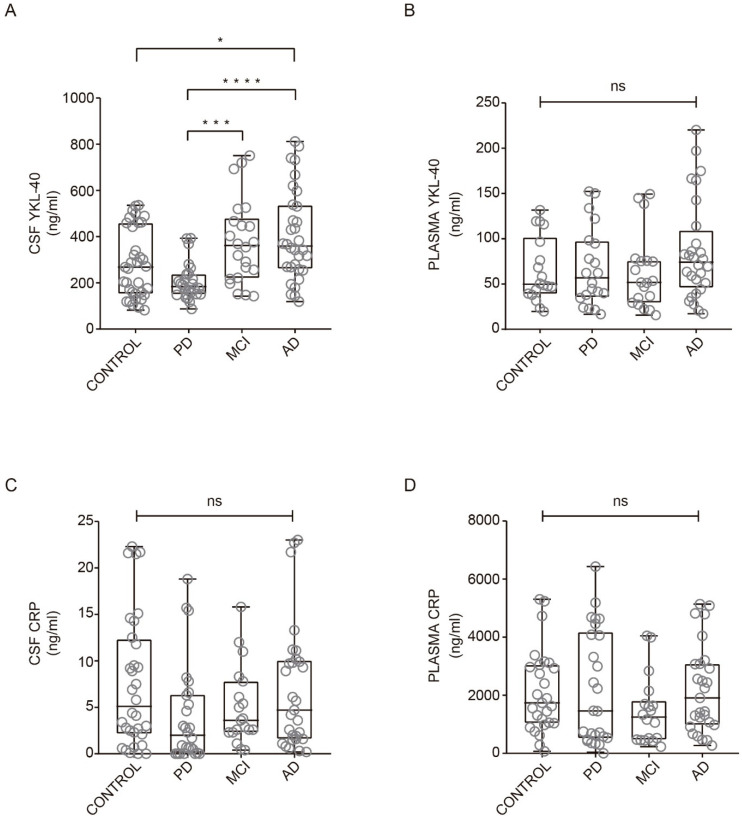
YKL-40 and CRP levels in CSF and plasma in different diagnostic groups. Box-and-whisker plots showing (**A**,**B**) YKL-40 and CRP levels (**C**,**D**) in CSF and plasma, respectively, across the diagnostic groups. Differences between groups were assessed using Kruskal–Wallis test followed by Bonferroni correction. * *p* < 0.05; *** *p* < 0.001; **** *p* < 0.0001. MCI, mild cognitive impairment; AD, Alzheimer’s disease dementia; PD, Parkinson’s disease. ns: non-significant.

YKL-40 and CRP levels across all clinical groups are illustrated in Figure 1. In CSF, YKL-40 levels were different among groups and were found to increase in AD dementia subjects compared with healthy controls (Figure 1A). No differences were found in YKL-40 levels between healthy controls and MCI or PD groups in CSF (Figure 1A). Nevertheless, a trend toward reduced levels was observed in PD patients, which were significantly lower compared to AD and MCI patient groups (Figure 1A). In plasma, YKL-40 levels remained unchanged across all clinical groups (Figure 1B).

A nonparametric trend test did not show any statistically significant rising tendency of CSF (*p* = 0.48) or plasma (*p* = 0.053) YKL-40 levels along with MCI or mild and moderate AD. When adjusting for age, sex, and APOE ε4 status, levels of CSF YKL-40 remained high in AD dementia patients when compared with controls (b = 125.5 ng/mL, 95% CI = 19.1 to 232.0 ng/mL, *p* < 0.05).

Regarding CRP levels in CSF and plasma, we did not find significant differences between healthy subjects and AD, MCI, and PD patients (Figure 1C,D). Our results are consistent with previous studies indicating no differences in CRP levels from CSF comparing healthy subjects and PD patients [26] or in serum CRP levels between patients with AD and healthy subjects [30].

**Figure 2:**
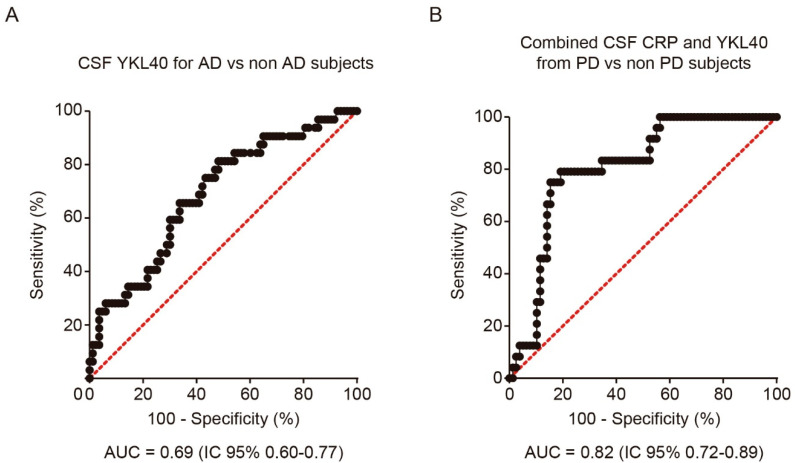
Receiver operating characteristic (ROC) analysis of YKL-40 and CRP levels in CSF. (**A**) ROC curve and its corresponding area under the curve (AUC) differentiating YKL-40 levels in CSF from AD patients and non-AD subjects including control subjects. (**B**) AUC differentiating the combination of YKL-40 and CRP levels in CSF from PD and non-PD patients. AUC, area under the curve; AD, Alzheimer’s disease dementia; PD, Parkinson’s disease.

In order to analyze the discriminative ability of both biomarkers for the diagnosis of PD and AD, we performed a logistic regression analysis and calculated the corresponding ROC curve for each CSF biomarker and diagnosis. CSF YKL-40 differentiated AD patients from the rest of the cohort, including PD, MCI, and healthy subjects, with 65.6% sensitivity and 66.3% specificity (AUC = 0.69, 95%CI = 0.58 to 0.80, cutoff point = 316.5 ng/mL) (Figure 2A). The combination with CSF CRP did not improve the performance. Nevertheless, for the diagnosis of PD, the combination of CSF YKL-40 and CRP yielded the best results, showing a moderate discriminative ability (AUC = 0.82, 95% CI =0.73 to 0.89, cutoff point of the model = 0.300), with 79.2% sensitivity and 82.1% specificity (Figure 2B).

### 3.3. Correlations between YKL-40 and CRP Levels in Plasma and CSF

**Figure 3:**
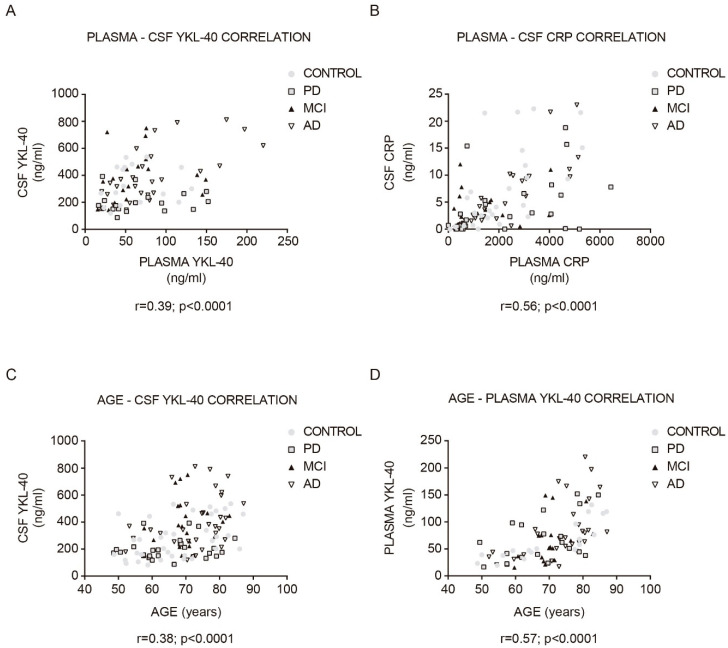
Correlation between YKL-40 and CRP levels in CSF and plasma, and between YKL-40 and age in the study cohort. Correlations between the expression levels of (**A**) YKL-40 and (**B**) CRP in CSF and plasma in the study cohort. Correlation between (**C**) CSF and (**D**) plasma YKL-40 and age within the diagnostic group. Correlations were examined with Pearson correlation test. MCI, mild cognitive impairment; AD, Alzheimer’s disease dementia; PD, Parkinson’s disease.

Both CSF YKL-40 (*r* = 0.39, *p* < 0.001; Figure 3A) and CRP (*r* = 0.56, *p* < 0.0001; Figure 3B) correlated significantly with their respective plasma concentrations in the whole cohort. The stronger positive correlation was found in AD patients (YKL-40: *r* = 0.69, CRP: *r* = 0.84).

In the whole cohort, plasma and CSF YKL-40 levels positively correlated with age (CSF YKL-40: *r* = 0.38, *p* < 0.0001; Figure 3C; plasma YKL-40: *r* = 0.57, *p* < 0.0001; Figure 3D). This correlation was especially stronger for the control group (CSF YKL-40: *r* = 0.46, *p* < 0.01; plasma YKL-40: *r* = 0.84, *p* < 0.0001). No statistically significant correlation with age was found in the plasma and CSF CRP analysis. Furthermore, the time since symptom onset did not correlate with any biomarker level in any group. Plasma and CSF YKL-40 and CRP levels did not differ by sex or by the presence of an APOE ε4 allele. 

### 3.4. YKL-40 Levels in AD Brain

**Figure 4:**
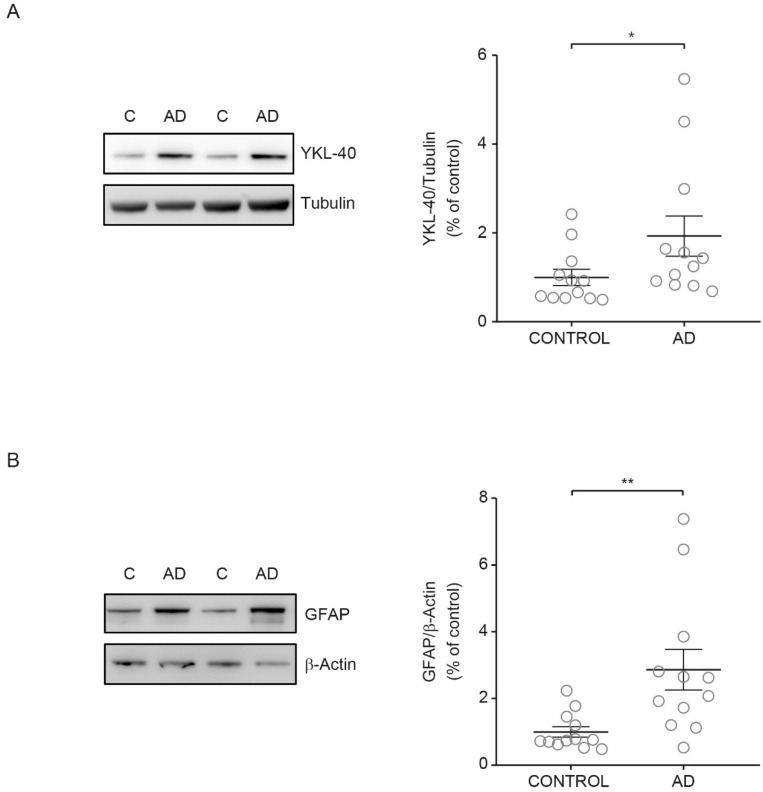
YKL-40 and GFAP levels in cerebral orbitofrontal cortex of AD patients and control group. Western blot analysis showing (**A**) YKL-40 and (**B**) GFAP in the cerebral orbitofrontal cortex of AD and control samples. Representative Western blots (left panels) and histograms with their densitometric analysis (right panels) are shown. Data are represented as the mean ± SEM. Differences between groups were assessed using Mann–Whitney test; * *p* < 0.05, ** *p* < 0.01.

Upon inflammation, YKL-40 is produced and secreted by many cells including vascular smooth muscle cells and macrophages [50]. In the brain, YKL-40 is mainly expressed in reactive astrocytes [20,25]. Thus, we investigated if the observed increase in YKL-40 levels in CSF from AD patients could be associated with higher YKL-40 levels in cerebral parenchyma. To explore this hypothesis, we examined the YKL-40 cellular levels in human brain tissue from AD patients and healthy subjects. Immunoblotting showed that YKL-40 levels in cerebral orbitofrontal cortex samples were significantly increased in AD patients compared with healthy subjects (Figure 4A). To determine if increased levels of YKL-40 in cerebral orbitofrontal cortex were associated with astrocyte reactivity, the levels of GFAP were also analyzed. Western blotting showed that GFAP levels were also higher in AD samples compared to those observed in control subjects (Figure 4B) in parallel with the observed rise in YKL-40 levels, proving that AD astrogliosis increases YKL-40 levels. 

## 4. Discussion

In this cross-sectional study, we showed a variable pattern of the inflammatory biomarkers YKL-40 and CRP in AD and PD patients. We confirmed that YKL-40 levels are significantly increased in CSF from AD patients compared to healthy controls, indicating an inflammatory response at the dementia stage. Such an increase was not seen in MCI or PD patients, where CSF YKL-40 levels remained unchanged. These results were also extended to the cerebral orbitofrontal cortex where we found that YKL-40 expression was augmented in AD patients, suggesting glial activation, thus corroborating our hypothesis. Another finding in this study was related to CRP levels in CSF and plasma. We found lower CRP levels in CSF from PD patients compared with other groups (AD, MCI, and healthy subjects), but this change did not reach statistical significance. Furthermore, we did not find evidence of significant alterations in plasma for YKL-40 or CRP. 

Inflammation is increasingly recognized as part of the pathology of neurodegenerative conditions, including AD and PD. Evidence proposes that neurodegeneration occurs in part because the CNS environment is affected by a cascade of events collectively named neuroinflammation [51]. Despite biomarkers of neuroinflammation being useful for monitoring disease diagnosis, progression, and response to therapy, accurate and reliable biomarkers for many neurological diseases are scarce. In recent years, the interest in new neuroinflammatory biomarkers has grown at early and symptomatic stages of these diseases. Blood and CSF are commonly used to monitor biomarkers of neuroinflammation, with many of them being the consequence of the CNS pathology. Some examples are the levels of cytokines and chemokines, the loss of blood–brain barrier integrity, and neuronal damage indicators [52]. 

Only a few studies have shown the possibility of analyzing YKL-40 levels in CSF and blood from patients with AD and predementia stages. One of these studies found that YKL-40 concentration in CSF from AD patients was significantly elevated compared to cognitively normal subjects, with an AUC = 0.88 pointing to the potential value of YKL-40 levels in CSF for AD diagnosis [53]. Increased YKL-40 levels were observed not only in AD dementia, but also in the prodromal phase of AD when compared to cognitively normal controls [54]. Similar observations were found in patients with AD, where YKL-40 concentration in CSF was increased in very mild and mild dementia subjects in comparison with cognitively normal individuals [16]. In our study, we found a trend of increased YKL-40 levels in CSF from MCI subjects compared with healthy controls, and this increase was evident in AD patients. However, the resulting AUC in our study was lower; thus, we propose that YKL-40 might only be a modest AD biomarker candidate.

Significantly increased *chitinase-3 like 3* (*CHI3L3*) mRNA expression, a mouse homolog of YKL-40, was found in brains of AD mice models when compared to age-matched controls [55]. Similarly, in autopsied human brain samples from pathologically confirmed AD subjects, *YKL-40* mRNA levels were significantly increased in comparison with nondemented controls [55]. Although there is no clear explanation regarding which factors modulate YKL-40 levels in AD, it has been suggested that elevated *YKL-40* expression and protein levels might result from increased astrocytic reactivity and release in brain [21]. It was shown that astrocytes in the close vicinity of amyloid plaques were immunoreactive for YKL-40, which confirms the involvement of this protein in the neuroinflammatory response to Aβ deposition [16]. It is known that insoluble Aβ aggregates may induce inflammatory reactions and activation of microglia, resulting in increased proinflammatory mediator production. The relationship between YKL-40 and amyloid-related pathways in AD development was further discussed [17,25]. It seems that the YKL-40 concentration in CSF may be linked to AD pathology, particularly astrogliosis. Indeed, it has been shown that *YKL-40* is expressed by reactive astrocytes GFAP+ in AD [25]. Thus, increased expression of *YKL-40* and protein levels in reactive astrocytes may be reflected in the CSF, indicating that astrocyte-associated metabolites may be utilized as potential biomarkers. Although data regarding elevated YKL-40 levels in CSF from early stages of AD are contradictory [16,17,22,23,24,54], our results support the increase in YKL-40 levels in CSF from AD subjects, as well as the increased astrocytic YKL-40 levels associated with astrocytosis.

Interestingly, we found that YKL-40 levels in CSF from PD patients were significantly lower compared with those levels in AD subjects suggesting that YKL-40, a marker of astroglial activation, is downregulated in PD. It was reported that YKL-40 levels were decreased in synucleinopathies when compared with tauopathies, suggesting that glial activation may be lower in brains from PD patients and other synucleinopathies in comparison with patients who have tauopathies or healthy controls [26,56]. These data may suggest that CSF YKL-40, as a marker of astroglial activation, is downregulated in PD. Despite astrocytes exerting protection against the inflammatory response in PD [57,58], astroglial dysfunction due to α-syn inclusions may occur simultaneously. In vitro evidence showed that astrocytes are able to efficiently degrade the α-syn aggregates from the extracellular space [59]. More recently, it was shown that primary rat astrocytes receive α-syn aggregates from neurons in mixed cell culture and efficiently transfer them from astrocyte to astrocyte [60]. It is possible that the increase in α-syn levels in astrocytes is a consequence of an endocytic mechanism upon high α-syn levels from the extracellular space, leading to the typical α-syn astrocytic inclusions in PD brains [61]. This accumulation could then lead to the dysregulation of other astrocytic functions, including YKL-40 production/secretion.

Our study yielded no significant changes for CRP levels in CSF or in plasma from AD and PD subjects, although others have described contradictory results [30,31,32,34]. Pathological studies have demonstrated that CRP is present in the senile plaques and neurofibrillary tangles in AD brains, suggesting that this protein may play a role in the neuropathological processes in AD [62,63,64]. In PD, aggregated α-syn can promote microglial activation and stimulate the secretion of inflammatory molecules, including CRP [65], thus evoking neuroinflammation [66].

CRP is primarily produced in the liver but is also generated in neurons to a lesser extent [41]. Such residual production of CRP in the CNS does not appear to contribute significantly to CSF levels [39].

In summary, our present study revealed a different inflammatory biomarker profile in individuals with AD and PD. CSF YKL-40 levels were significantly elevated in the AD group, and this increment corroborated the analysis of the YKL-40 protein levels in the cerebral orbitofrontal cortex from pathologically confirmed AD subjects. In PD individuals, plasma and CSF CRP and YKL-40 levels remained unchanged. Notwithstanding, we identified a moderate discriminative ability by combining both biomarkers in CSF for PD diagnosis. Together, our data support the involvement of both inflammatory proteins in the pathogenesis of neurodegenerative diseases.

## Figures and Tables

**Table 1:** Demographic and clinical data of participants.

	Control	PD	MCI	AD Dementia	*p* Value
*n*	37	30	22	34	NA
Sex (M/F)	22/15	17/13	7/15	13/21	ns
Age, mean (SD), y	68.18 (11.2)	66.39 (9.9)	69.40 (6.4)	73.53 (8.9) ^a^	<0.05
Age at onset, mean (SD), y	NA	61.48 (10.7)	66.53 (6.7)	70.44 (8.9) ^b^	<0.01
Years since onset, mean (SD), y	NA	3.89 (3.3)	2.87 (1.3)	3.09 (1.4)	ns
Hoehn & Yahr (1/2/3/4/5)	NA	11/11/6/2/0	NA	NA	NA
CDR (0.5/1/2/3)	NA	NA	22/0/0/0	0/25/9/0	NA
*APOEε* 4 carrier, No. (%)	1 ^c,d^	-	54	32.4	<0.0001

AD: Alzheimer’s disease; MCI: mild cognitive impairment. PD: Parkinson’s disease; n: number; F: female; ns: non-significant; y: year; M: male; SD: standard deviation; NA, not applicable; CDR: Clinical Dementia Rating. *p* value indicates statistical difference within the cohort1; -: not obtained data; ^a^ *p* < 0.05 vs PD; ^b^ *p* < 0.01 vs. PD; ^c^ *p* < 0.0001 vs. AD; ^d^ *p* < 0.0001 vs. PD.

**Table 2:** Demographic and clinical data of brain tissue donors.

	Control	AD
*n*	12	12
Sex (M/F)	6/6	6/6
Age, mean (SD)	73.25 (8.8)	76.33 (10.3)
Braak stage (*n*)	None: 7 Braak I: 3 Braak II: 2	Braak V: 9 Braak VI: 3

AD: Alzheimer’s disease; n: number; F: female. M: male; SD: standard deviation.

## Data Availability

The data obtained and presented in this study are available upon reasoned request from the corresponding author.
